# Differential Effects of Hormones on Cellular Metabolism in Keratoconus *In Vitro*

**DOI:** 10.1038/srep42896

**Published:** 2017-02-17

**Authors:** Tina B. McKay, Jesper Hjortdal, Henrik Sejersen, Dimitrios Karamichos

**Affiliations:** 1 Department of Cell Biology, University of Oklahoma Health Sciences Center, Oklahoma City, OK, 73104, USA; 2 Department of Ophthalmology, Aarhus University Hospital, Aarhus C DK-800, Denmark; 3 Department of Ophthalmology/Dean McGee Eye Institute, University of Oklahoma Health Sciences Center, Oklahoma City, OK, 73104, USA

## Abstract

Keratoconus (KC) is a corneal thinning disease with an onset commonly immediately post-puberty and stabilization by 40 to 50 years of age. The role of hormones in regulating corneal tissue structure in homeostatic and pathological conditions is unknown. Our group recently linked altered hormone levels to KC. Our current study sought to investigate and delineate the effects of exogenous hormones, such as androgen, luteotropin, and estrogen, on corneal stroma bioenergetics. We utilized our established 3D *in vitro* model to characterize the effects of DHEA, prolactin, 17β-estradiol on insulin-growth factor-1 and -2 (IGF-1, -2) signaling and metabolic function in primary corneal fibroblasts from healthy controls (HCFs) and KC patients (HKCs). Our data showed that exogenous DHEA significantly downregulated IGF-1 and its receptor in both HCFs and HKCs with HKCs showing consistently lower basal pentose phosphate flux. Prolactin caused no significant change in IGF-1 levels and an increase in IGF-2 in HKCs correlating with an increase in ATP and NADH levels. 17β-estradiol led to a significant upregulation in pentose phosphate flux and glycolytic intermediates in HCFs. Our results identified hormone-specific responses regulated in HKCs compared to HCFs revealing a novel role for hormones on bioenergetics in KC.

Sex hormones play a functional role in regulating growth and reproduction, systemic metabolism, and cellular differentiation and functionality^1^,^2^,^3^. Dehydroandrosterone (DHEA) is a steroid produced primarily by the adrenal gland, gonads, and brain that is readily converted to androgens or estrogens in a tissue-dependent fashion^4^,^5^ with circulating plasma levels ranging from 1–6 ng/mL^6^. The sulfated form of DHEA, dehydroandrosterone sulfate (DHEA-S), is present at much higher concentrations in circulation (450–3470 ng/mL) depending on age and sex^7^, which upon cellular uptake is de-sulfated and converted to steroid metabolites. DHEA-S supplementation has been shown to increase plasma levels of insulin growth factor-1 (IGF-1)^8^,^9^ with suggestions that depletions in DHEA may be linked to processes associated with aging^10^,^11^,^12^. Estrogens, estriol, 17β-estradiol (E2) and estrone, are produced by the ovaries in females and in small amounts by the prostate in males. Many of these hormones are known to regulate IGF-1 signaling directly during development and in pathological states, such as cancer^13^,^14^,^15^,^16^. IGF-1 is a pleiotropic growth factor whose production is stimulated by human growth factor serving as a key regulator of anabolic processes that promote tissue growth^17^,^18^. IGFs have been found to be essential growth factors during embryonic development mitigating a role in ventricular chamber heart development in mice^19^. Heterogeneous knockout of IGF-1R in mice increased mean lifespan by 26% than wild-type controls suggesting that IGF-1 flux may be involved in a complicated mechanism in the regulation of aging^20^. Localized IGF-1 production is suggested to play a fundamental role in regulating postnatal tissue growth compared to systemic flux^21^. In contrast, an overabundance of IGF-1 production due to elevated human growth hormone after puberty promotes the development of acromegaly, which results in overgrowth of connective tissue, skeletal aberrations, and cardiovascular defects^22^,^23^. Increased corneal thickness has also been identified in some acromegaly patients suggesting that overproduction of IGF-1 promoted by human growth hormone may promote stromal thickening within the eye as well^24^,^25^. Whether the effects of DHEA on corneal extracellular matrix (ECM) deposition are mediated via IGF-1 regulation is unknown.

Corneal thickness varies throughout development to adulthood and from person to person^26^. A major contributor to corneal thickness has been associated with altered hormone levels with varying effects on corneal thickness occurring during pregnancy^27^ and aging^28^. In females, corneal thickness has been shown to be greatest at ovulation and at the end of the menstrual cycle^29^,^30^. Furthermore, estrogen supplementation has been suggested to promote increased corneal thickness in post-menopausal women^31^. Hormones mediate changes in cell function via binding to their respective receptors with both the androgen receptor^32^,^33^ and estrogen receptor^34^ being expressed within the human cornea suggesting that hormones may influence corneal function directly. A published report identified that healthy corneal epithelium and conjunctival cells transcribe the genes for enzymes required for intracrine production of various hormones^35^, including 3β-hydroxysteroid dehydrogenase, which converts estrogens and androgens to activated forms via hydroxylation at carbon-17 of the steroid ring^36^. This study suggested that tissues of the anterior segment of the eye may be able to produce hormones *in situ* thereby influencing localized cellular signaling in an autocrine or paracrine fashion independent of systemic flux.

The common corneal dystrophy, Keratoconus (KC), is associated with significant thinning of the central cornea leading to severe visual defects with onset usually post-puberty and stabilization by the fourth to fifth decade of life^37^,^38^. KC has been reported to affect a higher predominance of males to females^39^,^40^. Our previous studies have indicated that systemic hormone levels are altered in KC patients compared to age- and gender-matched controls suggesting that systemic hormone flux may initiate a cascade of downstream signals that collectively may be responsible for KC development^41^. The KC onset at roughly 15 years of age, as well as the gender dependence and stabilization of the disease in middle age, support our proposed mechanism that a developmental change in a hormone-specific relationship may contribute to KC pathogenesis. In our current study, we investigated the effects of exogenous hormones in HCFs and HKCs in an attempt to delineate the role of hormones in KC using our 3D *in vitro* model. Our data revealed a novel mechanism of action of hormones in regulating IGF-1 expression and bioenergetics in corneal fibroblasts providing insight into possible causation and progression of corneal thinning in KC.

## Results

### Metabolic Response to DHEA

**Figure 1:**
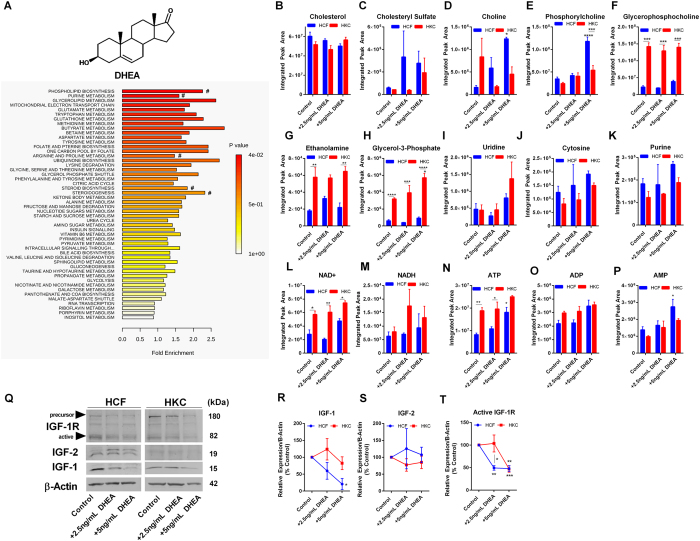
Effects of DHEA on metabolic pathways and IGF-1 expression in healthy human corneal fibroblasts (HCFs) and human keratoconus cells (HKCs). (**A**) Chemical structure of DHEA and enrichment pathways identified in HCFs by MetaboAnalyst following DHEA treatment. (# denotes pathways highlighted by metabolite breakdown). Quantitative determination of metabolite levels by targeted LC-MS/MS important in (**B**,**C**) steroid biosynthesis and metabolism, (**D**–**H**) phospholipid biosynthesis and signaling, and (**I**–**J**) pyridines, and (**K**) purine metabolism. (**L**–**P**) NAD+/NADH, ATP, ADP, and AMP flux in HCFs and HKCs detected by LC-MS/MS. (**Q**–**T**) Representative western blots and quantification of protein levels measured by densitometry showing a significant reduction in IGF-1 and its active receptor IGF-1R with no change in IGF-2 expression following DHEA treatment (2.5 ng/mL and 5 ng/mL). Western blots were converted to greyscale. Statistical significance determined by a two-way ANOVA. Experiments were performed in triplicate. Error bars represent standard error of the mean. *p < 0.05, **p < 0.01, ***p < 0.001, ***p < 0.0001. Note: controls were treated with vehicle (1% DMSO).

KC has been associated with altered cellular metabolism by HKCs with increased lactate production and elevated oxidative stress^42^. In order to determine if exogenous DHEA regulates cellular metabolism, we performed a pathway enrichment analysis of metabolites from control and DHEA-treated samples. The primary pathways enriched in HCFs following DHEA treatment involved lipid metabolism (phospholipid biosynthesis, glycerolipid metabolism, glycerol phosphate shuttle), protein metabolism (glutamate, tryptophan, methionine, arginine, aspartate, and tyrosine metabolism), and glucose metabolism (mitochondrial electron transport chain, citric acid cycle, glycolysis, pyruvate metabolism) (Fig. 1A). Cholesterol serves as a precursor to steroid-biosynthesis, as well as an essential structural component of the lipid bilayer. Cholesterol sulfate also functions as a source of steroid precursors, including DHEA-S and pregnenolone sulfate, and in addition, is a regulator of phosphatidylinositol 3-kinase and protein kinase C^43^. No change was found in basal cholesterol levels but a trend in increasing cholesterol sulfate in both HCFs and HKCs with DHEA treatment (Fig. 1B,C). Metabolites important in phospholipid biosynthesis and signaling, phosphorylcholine and choline, were significantly increased in HCFs (3.4-fold and 7.8–fold, respectively, p < 0.05) with DHEA treatment (5 ng/mL) with little change in HKCs (Fig. 1D,E). Glycerophosphocholine, ethanolamine, and glycerol-3-phosphate, important mediators involved in lipid signaling and lipogenesis, were increased in HKCs under all conditions compared to HCFs (p < 0.01, Fig. 1F–H). Furthermore, glycerol-3-phosphate, which serves as a precursor to many glycerophospholipids, was significantly increased with DHEA treatment (5 ng/mL) in HKCs highlighting lipid metabolism and phospholipid-mediated signaling as an important target of DHEA (1.8-fold, p < 0.05, Fig. 1H). Nucleic acid metabolism also showed an increasing trend with DHEA treatment in both cell types with elevated uridine and purine flux suggesting DHEA may modulate availability of nucleic acids required for gene transcription (Fig. 1I–K).

We further determined the levels of nicotinamide adenine dinucleotide (NAD+), nicotinamide adenine dinucleotide hydride (NADH), adenosine triphosphate (ATP), adenosine diphosphate (ADP), and adenosine monophosphate (AMP). We found a significant reduction in NAD+ in HCFs compared to HKCs in all conditions (p < 0.05) with a trend of increasing levels with the highest concentration of DHEA and no significant change in NADH levels (Fig. 1L,M). Furthermore, DHEA treatment (5 ng/mL) led to a significant increase in ATP (2.3-fold, p < 0.01) and AMP flux (2-fold, p < 0.05) with control HCFs having significantly lower levels of ATP compared to control HKCs (2.3-fold, p < 0.05) and no difference in ADP production suggesting that DHEA may contribute to altered energy production by affecting nucleotide availability or possibly glucose metabolism (Fig. 1N–P).

Since IGFs are one of the major regulators of metabolism^44^,^45^ and are known to be affected by hormone flux^17^,^46^, as well as play an important role in corneal epithelial wound healing^47^, we investigated whether the changes in metabolic regulation were related to altered protein expression of localized IGF-1, IGF-2, and the active receptor IGF-1R (Fig. 1Q–T). We found a significant reduction in IGF-1 production by 4.9-fold with 5 ng/mL DHEA treatment (p < 0.01) in HCFs with no significant reduction in HKCs (Fig. 1R). The expression of the alternative isoform, IGF-2, was not affected by DHEA treatment in either HCFs or HKCs (Fig. 1S). A reduction in the primary IGF-1 receptor, IGF-1R, was measured in both HCFs and HKCs by 2-fold (p < 0.01) with the most significant response at the high dose in HKCs suggesting that DHEA may directly regulate localized IGF-1R signaling by targeting both ligand and receptor expression (Fig. 1T). Flux of amino acids, glutamate, asparagine, and alanine, increased with DHEA treatment in HCFs with no effect in HKCs (Supplemental Fig. 1, p < 0.05). Our results suggest that DHEA downregulates IGF-1 leading to modulation of net protein synthesis or degradation in HCFs with the measured increase in amino acid flux correlating with increased catabolism. The lack of change in free amino acid flux in HKCs may be related to the sustained IGF-1 levels suggesting that ligand expression may be the limiting factor in regulating IGF-signaling compared to the active receptor, IGF-1R.

### DHEA and the Effects on Glycolysis, TCA, and PPP

**Figure 2:**
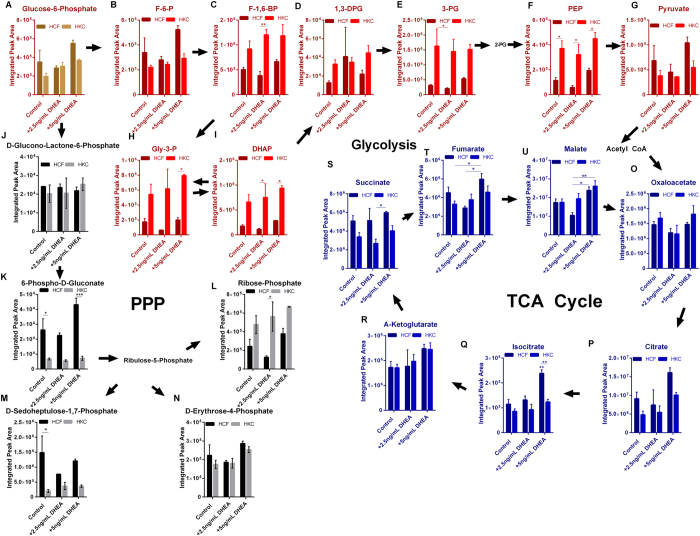
Effects of DHEA on glucose metabolism in HCFs and HKCs. (**A**–**I**) Glycolytic flux, (**J**–**N**) Pentose phosphate flux, and (**O**–**U**) TCA cycling in HCFs and HKCs following DHEA treatment (2.5 ng/mL and 5 ng/mL). Experiments were performed in triplicate, error bars represent standard error of the mean. Statistical significance was determined by ANOVA. *p < 0.05, **p < 0.01, ***p < 0.001. (Abbrev. F-6-P, fructose-6-phosphate; F-1,6-BP, fructose-1,6-bisphosphate; 1,3-DPG, 1,3-diphosphoglycerate; 3-PG, 3-phosphoglycerate; PEP, phosphoenolpyruvate; Gly-3-P, glyceraldehyde-3-phosphate; DHAP, dihydroxyacetone phosphate.) Note: controls were treated with vehicle (1% DMSO).

In order to determine the effects of DHEA on the major pathways involved in glucose metabolism, we evaluated metabolite flux in glycolysis, tricarboxylic acid cycle (TCA), and the pentose phosphate pathway (PPP). Surprisingly, no significant change in glycolytic flux was measured with exogenous DHEA in either cell type (Fig. 2A–I). However, HKCs showed increased flux of glycolytic intermediates, including fructose-1,6-bisphosphate (3-fold, p < 0.01), phosphoenolpyruvate (5-fold, p < 0.05), and dihydroxyacetone phosphate (6.7-fold, p < 0.05), compared to HCFs with DHEA treatment (2.5 ng/mL) (Fig. 2C,F,I). Isocitrate was significantly upregulated (2-fold, p < 0.01) in HCFs with DHEA treatment (5 ng/mL) with trends of increases in other TCA intermediates, including fumarate and malate (Fig. 2Q–U). Though HKCs showed increased basal glycolytic flux compared to HCFs, they showed no increase in TCA flux correlating with an increase in aerobic glycolysis in a Warburg-like phenomena^48^ with KC being previously associated with elevated lactate production^42^.

It has previously been reported that DHEA inhibits the PPP in human endometrial stromal cells^49^ and tumor cells^50^. The PPP pathway has also been identified by us as a target of the antioxidant, Quercetin, in HKCs with upregulation of glucose-6-phosphate, glyceraldehyde-3-phosphate, and erythrose-4-phosphate^51^. Treatment of HCFs and HKCs with 2.5 ng/mL and 5 ng/mL DHEA did not cause a significant reduction in PPP intermediates in our model (Fig. 2A,J–N). Interestingly, we measured a significant reduction in 6-phospho-D-gluconate and D-sedoheptulose-1,7-phosphate in control HKCs compared to control HCFs (2-fold and 3-fold, respectively, p < 0.05, Fig. 2K,M). Provided that 6-phospho-D-gluconate is converted to sedoheptulose-1,7-phosphate generating NADPH, the reducing agent responsible for converting oxidized glutathione to reduced glutathione, a reduction in this important substrate may relate to the increase in oxidative stress associated with KC.

Since we found that DHEA treatment influenced IGF-1 expression, we further evaluated the effects of DHEA on arginine metabolism, which can serve as a source of proline^52^ and polyamines^53^ affecting collagen assembly and cell survival, respectively. The urea cycle is a major regulator of arginine metabolism and occurs with the conversion of arginine to ornithine, which is then converted to citrulline and then to argininosuccinate. Urea is produced as a by-product following the conversion of arginine to ornithine. Following DHEA treatment (5 ng/mL), we observed a significant increase in carbamoyl production (2-fold, p < 0.05) in HCFs, with a slight increase in HKCs (Supplemental Fig. 2A). L-argininosuccinate was significantly elevated in HKCs with DHEA (2.5 ng/mL and 5 ng/mL, 2-fold and 3-fold, respectively, p < 0.01) compared to HCFs (Supplemental Fig. 2C). Arginine levels were increased slightly, though not significantly, with DHEA treatment suggesting that limiting reagents may restrict arginine flux (Supplemental Fig. 2D). Furthermore, both urea and ornithine levels were unchanged with DHEA treatment in either cell type (Supplemental Fig. 2E,F). The reduction in IGF-1 caused by DHEA suggests direct modulation of urea cycling in both HCFs and HKCs, which may affect the availability of precursors for proline and hydroxyproline biosynthesis involved in collagen assembly.

### Metabolic Response of Prolactin

**Figure 3:**
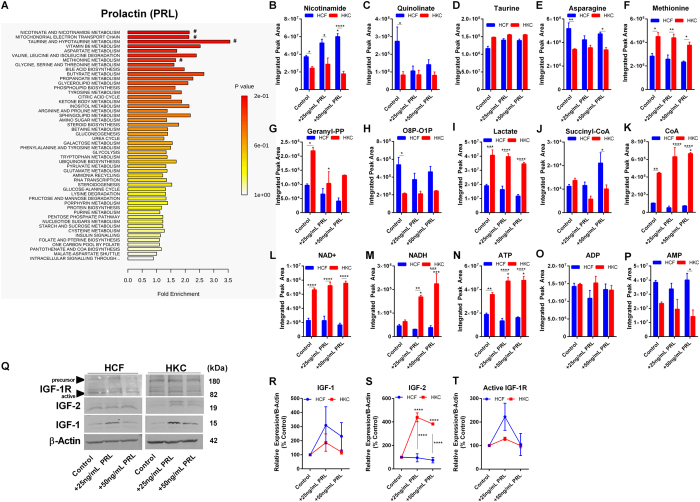
Effects of prolactin (PRL) (25 ng/mL and 50 ng/mL) on metabolic pathway and IGF-1 expression. (**A**) Pathway enrichment predicted by Metaboanalyst and metabolic breakdown based on (**A**,**B**) Nicotinate and quinolinate metabolism, and (**D**–**F**) free amino acid flux, (**G**) geranyl pyrophosphate, (**H**) oculose-8-phosphate-oculose-1-phosphate flux, (**I**–**K**) lactate and coenzyme A flux, (L-P) NAD+/NADH and ATP, ADP, and AMP flux. (Q-T) Representative western blots and quantification of bands by densitometry for IGF-1, IGF-2, and IGF-1R. Western blots were converted to greyscale. Statistical significance determined by a two-way ANOVA. Experiments were performed in triplicate. Error bars represent standard error of the mean. *p < 0.05, **p < 0.01, ***p < 0.001., ****p < 0.0001. Note: controls were treated with vehicle (purified water).

Prolactin has been shown to increase lactose and lipid production within the post-partum mammary gland^54^,^55^. Corneal curvature is known to be modulated during and following pregnancy suggesting a potential role for prolactin in regulating post-natal corneal structure^56^. In order to determine the effects of prolactin on bioenergetics in HCFs and HKCs, we measured metabolite flux and enrichment pathways following treatment with prolactin. We found that prolactin enriched pathways in HCFs associated with nicotinamide metabolism, mitochondrial electron transport chain, and butyrate metabolism (Fig. 3A). Nicotinamide was significantly upregulated 4.3-fold (p < 0.0001) in 50 ng/mL prolactin-treated HCFs compared to HKCs (Fig. 3B). Quinolinate is a product of tryptophan metabolism in the Kynurenine pathway with reports of elevated levels associated with neurodegenerative diseases, including Alzheimer’s and Huntington’s diseases, as well as contributing to increased oxidative stress^57^,^58^. We detected a significant reduction in quinolinate in control HKCs compared to HCFs with no modulation with prolactin treatment (3.3-fold, p < 0.05, Fig. 3C). Furthermore, we measured no significant change in free amino acid flux with prolactin treatment suggesting that protein synthesis and degradation are not affected by prolactin stimulation (25 ng/mL and 50 ng/mL) (Supplemental Fig. 3). Geranyl pyrophosphate (geranyl-PP), a metabolite important in mediating lipid biogenesis serving as a precursor in terpenoid biosynthesis^59^, was significantly upregulated in control HKCs (2.3-fold, p < 0.05) compared to HCFs with a modest reduction in HKC levels with prolactin treatment (25 ng/mL) (1.6-fold, p < 0.05, Fig. 3G). Octulose-8-phosphate and octulose-1-phosphate (O8P-O1P) are important in L-PPP flux in eukaryotic cells^60^ and were found to elevated in HCFs (2.5-fold, p < 0.05) compared to HKCs with little change following prolactin treatment (Fig. 3H).

We have previously reported elevated lactate production by HKCs *in vitro*^42^ and found no significant modulation by prolactin in this study with HKCs maintaining significantly higher lactate production compared to HCFs showing no improvement in the oxidative tendency of HKCs (2-fold, p < 0.001, Fig. 3I). Co-enzyme A and succinyl-CoA were upregulated slightly in 25 ng/mL prolactin-treated HCFs compared to HKCs (2-fold, p < 0.05) with HKCs showing overall higher levels of free coenzyme A with 50 ng/mL prolactin significantly increasing these levels in HKCs only (1.5-fold, p < 0.05, Fig. 3J,K). The availability of CoA levels would likely affect TCA flux given the requirement of this substrate in the biosynthesis of acetyl CoA, while the reduced levels in HKCs correlate with higher lactate production and glycolytic flux.

We further determined if prolactin affected production of high-energy biomolecules. Interestingly, though HKCs showed lower nicotinamide levels, NAD+ levels were upregulated in HKCs control, 25 ng/mL and 50 ng/mL prolactin-treated samples (2.9-fold, 3.2-fold, and 4.6-fold, respectively, p < 0.0001) with an increase in NADH production by 2.6–fold and 3.5-fold (p < 0.05 and p < 0.001, respectively, Fig. 3L,M). Correlating with this increase in NADH production by HKCs, ATP levels also increased 1.3–fold (p < 0.05) with prolactin treatment with no such increase detected in HCFs. In agreement with the DHEA experiments (Fig. 1N), basal ATP flux was higher in control HKCs compared to HCFs with little change in ADP and AMP levels (Fig. 3N–P).

In order to determine if the effects on NADH and ATP production in HKCs were attributed to altered IGF-signaling, we measured the protein expression of IGF-1, IGF-2, and the receptor IGF-1R (Fig. 3Q). Compared to the effects of DHEA, we found little modulation of IGF-1 or IGF-1R expression by prolactin but found a significant increase in IGF-2 production by HKCs (3.5-fold, p < 0.01) with no effects in HCFs (Fig. 3Q–T). Our results agree with previous reports that prolactin mediates changes primarily through IGF-2 in breast tissue^61^ and suggest a potential link to the effects of prolactin in regulating post-partum corneal structure via IGF-2 with higher responsiveness by HKCs.

### Prolactin and the Effects on Glycolysis, TCA, and PPP

**Figure 4:**
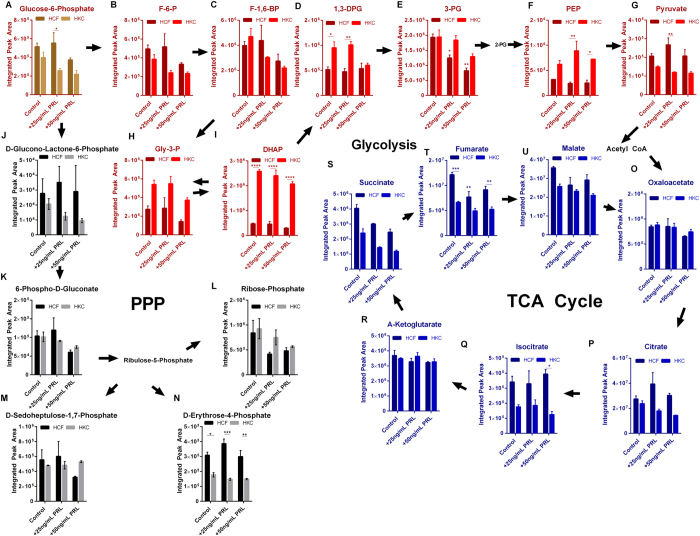
Effects of prolactin (PRL) on glucose metabolism in HCFs and HKCs. (**A**–**I**) Glycolytic flux, (**J**–**N**) Pentose phosphate flux, and (**O**–**U**) TCA cycling in HCFs and HKCs following PRL treatment (25 ng/mL and 50 ng/mL). Experiments were performed in triplicate, error bars represent standard error of the mean. Statistical significance was determined by ANOVA. *p < 0.05, **p < 0.01, ***p < 0.001, ****p < 0.0001. (Abbrev. F-6-P, fructose-6-phosphate; F-1,6-BP, fructose-1,6-bisphosphate; 1,3-DPG, 1,3-diphosphoglycerate; 3-PG, 3-phosphoglycerate; PEP, phosphoenolpyruvate; Gly-3-P, glyceraldehyde-3-phosphate; DHAP, dihydroxyacetone phosphate.) Note: controls were treated with vehicle (purified water).

In order to determine if exogenous prolactin resulted in changes in glucose metabolism, we investigated glycolytic, TCA, and PPP flux in HCFs and HKCs (Fig. 4). 3-Phosphoglycerate (3-PG) flux decreased 2-fold (p < 0.05) in HCFs with prolactin treatment (25 ng/mL and 50 ng/mL) (Fig. 4E) with little effect on the other glycolytic intermediates (Fig. 4A–I). Both phosphoenolpyruvate (PEP) and dihydroxyacetone phosphate (DHAP) were upregulated in all HKC samples compared to HCFs again correlating with increased glycolytic activity in HKCs with little influence by prolactin (Fig. 4F,I, p < 0.05). TCA flux showed higher levels of the intermediates, fumarate, isocitrate, and succinate in HCFs with and without prolactin treatment with reductions in levels following prolactin treatment (Fig. 4Q,S,T). While PPP flux was consistently higher in HCFs compared to HKCs with elevated erythtrose-4-phosphate (Fig. 4N), prolactin did not modulate PPP flux in either cell type (Fig. 4J–N). Furthermore, little effect occurred in amino acid flux (Supplemental Fig. 3) which agreed with no change in IGF-1 or IGF-1R levels induced by prolactin (Fig. 3Q) suggesting that the downstream effects of altered IGF-2 levels may not contribute to changes in protein synthesis and degradation or significant variations in glucose metabolism.

### Metabolic Response of E2

**Figure 5:**
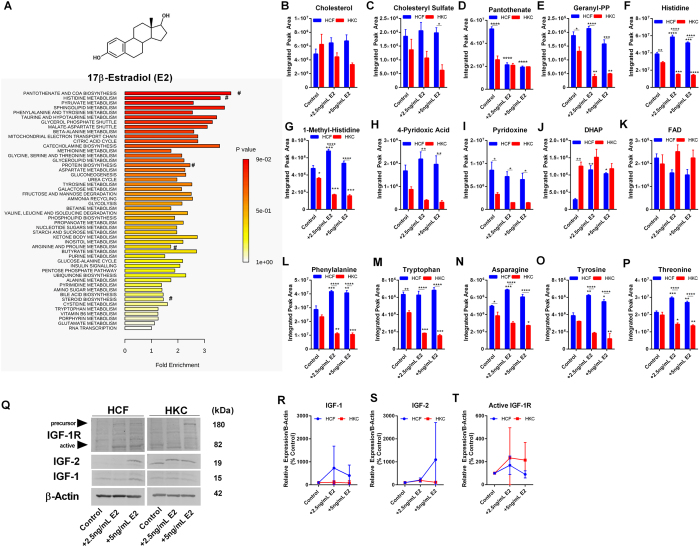
Effects of 17β-estradiol (E2) (2.5 ng/mL and 5 ng/mL) on metabolic pathways and IGF-1 expression in HCFs and HKCs. (**A**) Chemical structure of E2 and enrichment pathways identified in HCFs by MetaboAnalyst. (#denotes pathways highlighted by metabolite breakdown). Quantitative determination of metabolite levels by targeted LC-MS/MS important in (**B**–**C**) steroid biosynthesis and metabolism, (**D**,**E**) pantothenate and geranyl pyrophosphate flux, (**F**,**G**) histidine metabolism, (**H**,**I**) Vitamin B6 metabolism, and (**J**,**K**) glycolytic and flavone flux, and (**L**–**P**) amino acid flux with increasing E2 concentrations detected by LC-MS/MS. (**Q**–**T**) Representative western blots and quantification of protein levels measured by densitometry. Western blots were converted to greyscale. Statistical significance determined by a two-way ANOVA. Experiments were performed in triplicate. Error bars represent standard error of the mean. *p < 0.05, **p < 0.01, ***p < 0.001, ****p < 0.0001. Note: controls were treated with vehicle (1% DMSO).

Estrogens are known to modulate survival and proliferation of cells^62^ and promote corneal wound healing *in vitro*^63^. In order to determine if E2 influenced cellular metabolism in a similar manner as DHEA or prolactin, we investigated the effects of increasing concentrations of E2 on metabolic pathways and amino acid flux in HCFs and HKCs in our 3D *in vitro* model. Enriched metabolites associated with E2 treatment in HCFs involved pantothenate biosynthesis, histidine metabolism, and protein biosynthesis (Fig. 5A). Effects of E2 on lipid metabolism differed from DHEA-treated constructs with significant reductions in cholesterol sulfate and geranyl-pentaphosphate levels in HKCs (2-fold and 2.6-fold, respectively, p < 0.05), but not in HCFs, with little change in basal cholesterol levels (Fig. 5B,C). Pantothenate is functionally important in coenzyme A biosynthesis^64^ and may play a role in regulating reactive oxygen species flux in dermal fibroblasts^65^. Our results show that E2 reduced pantothenate levels in HCFs by 2.5–fold (p < 0.0001) with HKCs showing reduced levels even at basal conditions (Fig. 5E). Histidine was differentially regulated as well between HCFs and HKCs with E2 treatment (5 ng/mL) increasing it by 1.3-fold (p < 0.001) in HCFs and reducing it by 2–fold (p < 0.001) in HKCs (Fig. 5F). This trend was also seen with 1-methyl-histidine, a precursor important in histidine metabolism, with E2 treatment (2.5 ng/mL) increasing levels 1.4–fold (p < 0.001) in HCFs and reducing 2–fold (p < 0.001) in HKCs (Fig. 5G). Pyridoxine, as a form of Vitamin B6, functions as a cofactor for trans-sulfuration reactions with deficiencies associated with seizures^66^,^67^. Pyridoxine and 4-pyridoxic acid were both downregulated in HKCs with all treatments potentially identifying altered Vitamin B6 metabolism associated with KC (p < 0.01 and p < 0.05, respectively, Fig. 5H,I). Interestingly, E2 promoted upregulated DHAP levels in HCFs (3.7-fold, p < 0.01) suggesting modulations in glycolytic flux with no change in flavone levels (FAD) (Fig. 5J,K). In order to determine if amino acid flux was affected by E2 treatment (Supplemental Fig. 4), we quantified the expression of the following amino acids: phenylalanine, tryptophan, asparagine, tyrosine, and threonine (Fig. 5L–P). Significant increases were observed in the aromatic amino acids, phenylalanine and tyrosine in HCFs (1.4-fold and 1.4–fold, respectively, p < 0.01) with an inverse effect in HKCs (down 2.2–fold and 2.7–fold, p < 0.01) suggesting an increase in anabolic processes in HKCs, including protein biosynthesis, thereby promoting lower availability of amino acids with E2 treatment (5 ng/mL). In order to determine if the effects of E2 on amino acid metabolism were due to altered IGF signaling, we measured protein levels of IGF-1, IGF-2, and IGF-1R (Fig. 5Q). We found slight, but insignificant, changes in both IGF-1 and IGF-2 in HCFs with little effect in HKCs suggesting that E2 did not modulate energy production via the IGF-axis (Supplemental Fig. 5).

### E2 and the Effects on Glycolysis, TCA, and PPP

**Figure 6:**
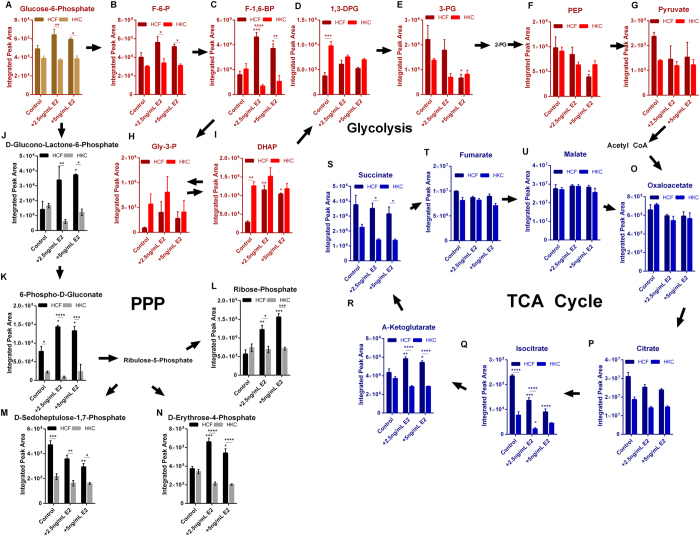
Effects of 17β-estradiol (E2) on glucose metabolism in HCFs and HKCs. (**A**–**I**) Glycolytic flux, (**J**–**N**) Pentose phosphate flux, and (**O**–**U**) TCA cycling in HCFs and HKCs following 17β-estradiol (E2) treatment (2.5 ng/mL and 5 ng/mL). n = 3, error bars represent standard error of the mean. Statistical significance was determined by ANOVA. *p < 0.05, **p < 0.01, ***p < 0.001, ****p < 0.0001. (Abbrev. F-6-P, fructose-6-phosphate; F-1,6-BP, fructose-1,6-bisphosphate; 1,3-DPG, 1,3-diphosphoglycerate; 3-PG, 3-phosphoglycerate; PEP, phosphoenolpyruvate; Gly-3-P, glyceraldehyde-3-phosphate; DHAP, dihydroxyacetone phosphate.) Note: controls were treated with vehicle (1% DMSO).

We sought to further define the effects of E2 on glucose metabolism and quantified metabolite flux in glycolysis, TCA, and PPP cycles (Fig. 6). As previously shown in Fig. 5J, we measured an increase in glycolytic flux with increased dihydroxyacetone phosphate in HKCs with E2 treatment (2.5 ng/mL and 5 ng/mL) leading to an increase in DHAP in HCFs (4.1-fold and 3.7–fold, respectively, p < 0.05, Fig. 6I), as well as elevated fructose-1,6-bisphopshate (F-1,6-BP) (2.9-fold and 2.3-fold, respectively, p < 0.05, Fig. 6C). Phosphoenolpyruvate (PEP) levels in control HKCs in this set of experiments were at similar levels as HCFs likely due to vehicle differences with E2 treatment (5 ng/mL) significantly reducing PEP in HCFs (2.5-fold, p < 0.05, Fig. 6F). Similar to the previous experiments showing reduced TCA intermediates in control HKCs compared to HCFs, stimulation with 2.5 ng/mL and 5 ng/mL E2 caused a significant reduction in isocitrate (1.7-fold and 2.6-fold, respectively, p < 0.001) with slight increases in α-ketoglutarate (p < 0.05) in HCFs and a 3.5-fold reduction in isocitrate in HKCs (p < 0.05, Fig. 6Q,R).

The most significant effect of E2 on glucose metabolism occurred in the PPP flux (Fig. 6J–N). E2 treatment (5ng/mL) led to a significant increase in glucono-lactone-6-phosphate (2.7-fold, p < 0.05), 6-phospho-D-gluconate (1.7-fold, p < 0.05), ribose-phosphate (2.8-fold, p < 0.001), and D-erythrose-4-phosphate (1.5-fold, p < 0.05) in HCFs, but not HKCs (Fig. 6J–L,N). The observed increase in PPP flux induced by E2 stimulation suggests that estrogens may play a direct role in regulating the alternative glucose shunt favoring PPP conversion over glycolysis.

## Discussion

Hormones are known to influence systemic metabolic rate by altering sugar and lipid uptake^68^,^69^. Various cellular processes, including survival and differentiation, proliferation and protein degradation, and secretion and assembly of the ECM are dependent on the energy-status of the cell^70^,^71^,^72^. It is still unclear what role bioenergetics play in regulating corneal keratocyte function and how alterations may contribute to cellular responses during wounding or pathologically ECM thinning as occurs in KC. In our study, we found significant reduction in basal PPP flux in control HKCs compared to HCFs. The sex hormones, DHEA and E2, require a lipophilic vehicle with utilization of dimethylsulfoxide (DMSO) in our study, while prolactin is a water-soluble protein. The differences in PPP metabolite flux, involving 6-phospho-D-gluconate, sedoheptulose-1,7-phosphate, and erythrose-4-phosphate, in control HCFs and HKCs depending on the presence of DMSO suggested that PPP flux is sensitive to the presence of a pro-oxidant vehicle though the trend of reduced PPP flux persisted in the HKC samples. Since PPP is known as a major regulator of antioxidant capacity via the production of NADPH which functions to reduce oxidized glutathione to the active form^73^, our data suggests that reduced pentose phosphate cycling in HKCs may be related to the inherent increased oxidative stress reported in a number of studies^42^,^74^,^75^,^76^. The PPP also produces glycolytic intermediates as a by-product, including glyceraldehyde-3-phosphate and fructose-6-phosphate, which serve to sustain glycolysis. In our studies, we found that HKCs maintained elevated glycolytic flux that correlated with increased lactate production, as previously reported^42^, suggesting that the reduction in PPP intermediates does not reduce the overactive glycolytic pathway present in HKCs.

Since KC is a disease with a post-puberty onset when endocrine function and sex hormone production is significantly modulated, our study focused on the role of exogenous hormones in regulating metabolic function within corneal fibroblasts, in self-assembled constructs, to determine if KC-derived cells exhibit an altered response. The use of fibroblasts compared to the more quiescent keratocyte natively found within the corneal stroma was required in long-term culture conditions where cells must be grown in the presence of serum. This 3D *in vitro* model has been a useful tool to determine pathological differences between HCFs and HKCs^42^,^77^. Characterization of long-term 3D *in vitro* cultures using corneal fibroblasts has been published in other studies evaluating the expression of proteoglycans and extracellular matrix structure at varying time points^78^,^79^.

**Figure 7:**
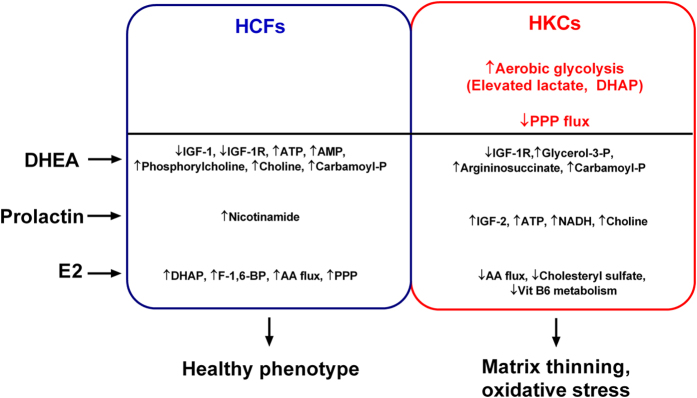
Summary of metabolic regulation induced by exogenous hormones: DHEA, prolactin, and E2 in HCFs and HKCs. A number of studies have associated mitochondrial dysfunction with KC^80^,^93^,^94^ and altered androgen and estrogen levels in KC patients^41^. Our previous studies^42^,^95^ and current work support increased lactate production in HKCs compared to HCFs correlating with upregulated aerobic glycolysis. We posit that a differential response to each hormone is detected in HKCs and HCFs due to this aberrant basal metabolism in HKCs.

A number of studies have identified increased oxidative stress, mutations in mitochondrial genes, and inflammatory protein expression in KC-derived cells *in vitro*^41^,^42^,^80^,^81^,^82^ and *ex vivo*^41^,^83^,^84^,^85^. We posit that oxidative stress promoted by altered androgen or estrogen levels during post-pubescence may be central to the altered metabolic function observed in HKCs. Our proposed mechanism of KC development focuses on early-age hormone levels and their role in regulating metabolic function, oxidative stress, and ultimately matrix thinning (Fig. 7). Our results from this study identified a regulatory role of exogenous hormones on localized IGF-1 production and the downstream effects on bioenergetics and metabolic function in corneal fibroblasts. We found that exogenous DHEA promoted downregulation of IGF-1 and the active IGF-1R receptor expression which resulted in an increase in select free amino acids. Given the role of choline and phosphorylcholine in regulating protein kinase C signaling, the increase in these metabolites in HCFs with DHEA treatment suggests that this androgen may modulate calcium signaling and a number of downstream pathways related to lipid-mediated signaling as well^86^.

While DHEA reduced IGF-1 and IGF-1R expression in both HCFs and HKCs, E2 did not significantly modulate expression of these growth factors. However, E2 stimulated a strong metabolic response with elevated glycolytic and PPP fluxes in HCFs suggesting bioenergetics are modulated by estrogen independent of the IGF-1 axis. In contrast, the luteotropin prolactin significantly increased IGF-2 expression only in HKCs, while having a modest effect on glucose metabolism and ATP production. Previous studies have shown that IGF-2 is the primary isoform responsible for prolactin-induced morphogenesis within breast tissue^61^. Our results support the hypothesis that prolactin modulates IGF-2 protein expression with the significant response occurring in our study in HKCs with no change in IGF-1 or IGF-1R expression. The IGF-1R is known to heterodimerize with the insulin receptor^87^ suggesting that changes in expression of IGF-1R may affect responses to insulin as well.

Though IGF-2 levels were significantly modulated in HKCs and not HCFs, glycolytic flux was slightly reduced in HCFs with no change in the disparity between the elevated levels in HKCs even at basal conditions. These results suggest that though DHEA, prolactin, and E2 influence cellular bioenergetics, the inherent defects that drive elevated glycolytic flux in HKCs are unable to be overcome with hormone stimulation at the physiological levels utilized in this study. Furthermore, our study revealed a novel finding that exogenous DHEA may contribute to KC development or progression by reducing localized production of IGF-1 and autocrine or paracrine signaling contributing to altered metabolic function. The increase in carbamoyl phosphate and argininosuccinate, key metabolites involved in the urea cycle, suggests that arginine and polyamine flux in HKCs may be more sensitive to DHEA-induced modulation of collagen precursors, perhaps leading to ECM thinning, a trademark of KC. In contrast, HKCs showed less sensitivity to E2 treatment compared to HCFs with dramatic increases in glycolysis and PPP flux. These results highlight a hormone-specific response in healthy versus KC-derived cells which may be a factor related to the altered basal metabolism observed in HKCs. Further studies are needed to elucidate the effects of hormones in regulating genes associated with matrix deposition and mitochondrial function in KC in order to determine if the sex chromosomes regulate the cellular responses to exogenous hormones *in vitro*.

## Materials and Methods

### Isolation of Primary Corneal Fibroblasts

The ethics committee of the University of Oklahoma Health Sciences Center and Aarhus University Hospital approved tissue collection (IRB protocols #3450 and #1-10-72-77-14, respectively) with written informed consent obtained from patients. All samples were de-identified prior to analysis. This study met the tenets of the Declaration of Helsinki. Cadaver tissue was provided by NDRI (National Disease Research Interchange). KC corneas were obtained from clinical collaborators immediately following corneal transplantation. Inclusion/exclusion criteria for healthy controls required absence of ophthalmic disease, diabetes, or infectious conditions. KC patients required diagnosed by an ophthalmologist for inclusion and absence of other corneal diseases. KC patients who had previously undergone collagen crosslinking were excluded from study. Donor information for HCFs utilized in this study: N19 (63 y/o male), N23 (21 y/o male), and N4 (53 y/o, male). Donor information for HKCs utilized in this study: DM1 (44 y/o female), WU1 (62 y/o female), and WU2 (34 y/o male). We isolated both HCFs and HKCs as previously described^77^,^88^. Briefly, the corneal epithelium and endothelium were removed using a surgical scalpel. The corneal stroma was isolated, washed in sterile PBS, and cut into small pieces (2 × 2 × 2 mm) and placed into flasks and allowed to adhere to the flask surface. Eagle’s Minimum Essential Media (EMEM) containing antibiotic/antimycotic (Anti/Anti, Life Technologies, Grand Island, NY) and 10% fetal bovine serum (Atlanta Biologicals, Flowery Branch, GA) were added to flask. Explants were grown 2-4 weeks at 37 °C/5% CO_2_/95% relative humidity until cells migrated from the tissue section into the flask. Cells were then isolated following trypsinization and subcultured or frozen using standard cryoprotective protocols.

### 3D *In Vitro* Model

The 3D *in vitro* model has been described extensively^77^,^88^. Briefly, primary corneal fibroblasts were seeded at 10^6^ cells/well in polycarbonate transwell plates (Corning Costar, Charlotte, NC) containing 1.5 mL of EMEM/10% FBS/anti-anti media in both the top and bottom wells. Post t = 24 hours following seeding, constructs were stimulated with a stable Vitamin C derivative (0.5 mM 2-O-α-D-glucopyranosyl-L-ascorbic acid, American Custom Chemicals Corporation, San Diego, CA) in the media following filter sterilization. Media containing hormones were freshly prepared and sterilized prior to addition to constructs: (2.5 ng/mL and 5 ng/mL) trans-dehydroandrosterone (Sigma Aldrich, St. Louis, MO), (2.5 ng/mL and 5 ng/mL) 17β-estradiol (Sigma Aldrich), and (25 ng/mL and 50 ng/mL) human prolactin (Sigma Aldrich). DHEA and 17β-estradiol were dissolved in sterile DMSO. 1% of DMSO was added to culture media. Lyophilized prolactin was dissolved in sterile distilled/deionized water. Media was changed every other day for the entire 4 week period, as previously described^77^.

### Metabolite Extraction and Targeted Mass Spectrometry

Constructs were isolated at the end of week four for metabolite isolation. As previously described^42^,^89^, metabolites were isolated using ice-cold (−80 °C) MeOH in water on dry ice, incubated, centrifuged, dried, and repeated two times. Dried pellets were stored at −80 °C until shipped on dry ice and analyzed. Metabolomics analysis was completed at Beth Israel Deaconess Medical Center mass spectrometry core facility. HPLC-grade water was used to dissolve pellets and analyzed for quantification by LC-MS/MS in a hybrid 5500 QTRAP triple quandrupole mass spectrometer (AB/SCIEX). MultiQuant 2.1 was used to quantify label-free metabolites. MetaboAnalyst 3.0 software was used to determine enrichment pathways, as previously described^90^,^91^. Heat maps were developed using the NCIminer server as previously reported by Weinstein *et al*.^92^ to generate a one matrix clustered image map with quantile binning.

### Protein Isolation and Western Blot

Constructs were collected, washed 2X with PBS, and total protein was isolated using 1X radioimmunoprecipitation assay (RIPA) buffer (50 mM Tris pH 8, 150 mM NaCl, 1% Triton X-100, and 0.1% SDS) containing a protease inhibitor cocktail (Sigma Aldrich). Cell lysates were centrifuged at 4 °C to precipitate insoluble debris, and the supernatant was isolated and subjected to a Pierce BCA Protein assay (ThermoScientific, Rockford, IL) for protein concentration determination. Western blot was performed using a gradient Novex 4–20% Tris-glycine gel (Life Technologies, Carlsbad, CA) electrophoresed at 135–140 V for 1.5 hours, and transferred onto a nitrocellulose membrane at 100 V for 1 hour on ice. Blots were blocked in 5% dry milk (Great Value, Bentonville, AR) or 5% BSA (Fisher, Fair Lawn, NJ) for 1 hour at room temperature with shaking. Anti-human rabbit primary antibodies were incubated with membrane overnight at 4 °C with rocking at concentrations of 1:500–1:1000: β-actin (Abcam, ab82227, Cambridge, MA), IGF-1 (Abcam, ab9572, Cambridge, MA), IGF-1R (Abcam, ab131476, Cambridge, MA), IGF-2 (Abcam, ab9574, Cambridge, MA). A fluorescent secondary antibody was used at a concentration of 1:1000-1:2000: Alexafluor 568 donkey anti-rabbit (Life Technologies, Eugene, OR). Detection and quantification of bands was measured using the ChemiDoc It2 Imager (UVP, Upland, CA) using densitometry with background subtraction and converted to grey scale.

### Statistical Analysis

A two-way ANOVA with multiple comparison analysis was performed in GraphPad Prism to determine significance with p < 0.05 considered statistically significant. Error bars represent standard error of the mean. The n numbers are listed in the appropriate figure legends.

## Additional Information

**How to cite this article:** McKay, T. B. *et al*. Differential Effects of Hormones on Cellular Metabolism in Keratoconus *In Vitro. Sci. Rep.* **7**, 42896; doi: 10.1038/srep42896 (2017).

**Publisher's note:** Springer Nature remains neutral with regard to jurisdictional claims in published maps and institutional affiliations.

## Supplementary Material

Supplementary Information
