# Supplementary material for: Differential Effects of Hormones on Cellular Metabolism in Keratoconus In Vitro
Source: Sci Rep. 2017 Feb 17;7:42896. doi: 10.1038/srep42896 (PMC5314412; doi:10.1038/srep42896)
Supplement: Supplementary Information [file srep42896-s1.pdf]

Differential Effects of Hormones on Cellular Metabolism in Keratoconus *In Vitro*

Tina B McKay[1], Jesper Hjortdal[2], Henrik Sejersen[2], Dimitrios Karamichos[1,3,]*

[1] *Department of Cell Biology, University of Oklahoma Health Sciences Center, Oklahoma City, OK, 73104, USA*

[2] *Department of Ophthalmology, Aarhus University Hospital, Aarhus C DK-800, Denmark.*

[3] *Department of Ophthalmology/Dean McGee Eye Institute, University of Oklahoma Health Sciences Center, Oklahoma City, OK, 73104, USA*

* **Corresponding author**: Dimitrios Karamichos, Ph.D., Department of Ophthalmology/Dean McGee Eye Institute, University of Oklahoma Health Sciences Center, Oklahoma City, OK 73104, USA. Tel.: +1 405 271 4019; fax: +1 405 271 8128; e-mail: dimitrios-karamichos@ouhsc.edu

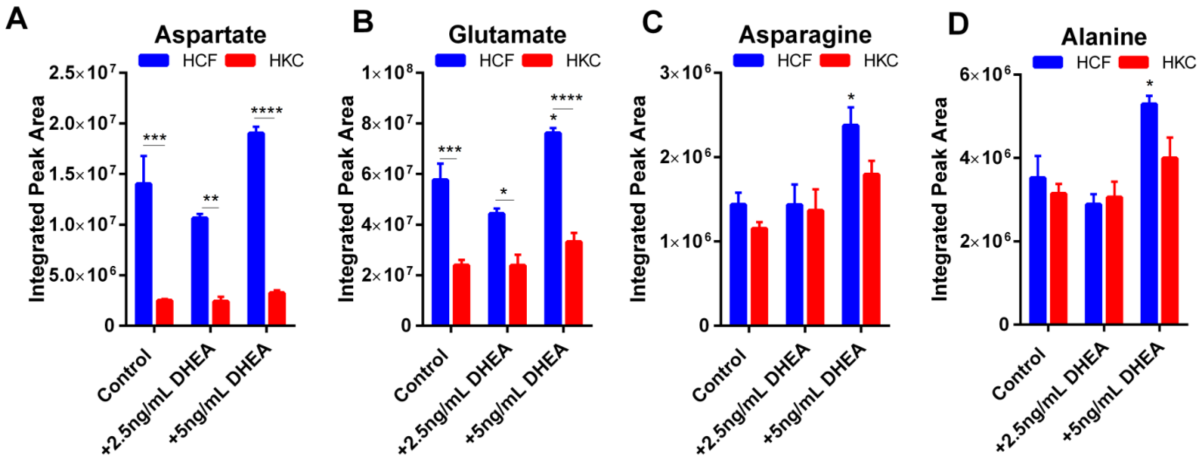

Supplemental Figure 1. Amino acid flux in HCFs and HKCs with increasing concentrations of DHEA (2.5ng/mL and 5ng/mL). Glutamate, asparagine, and alanine significantly increase with DHEA treatment (5ng/mL) in HCFs, but not HKCs.

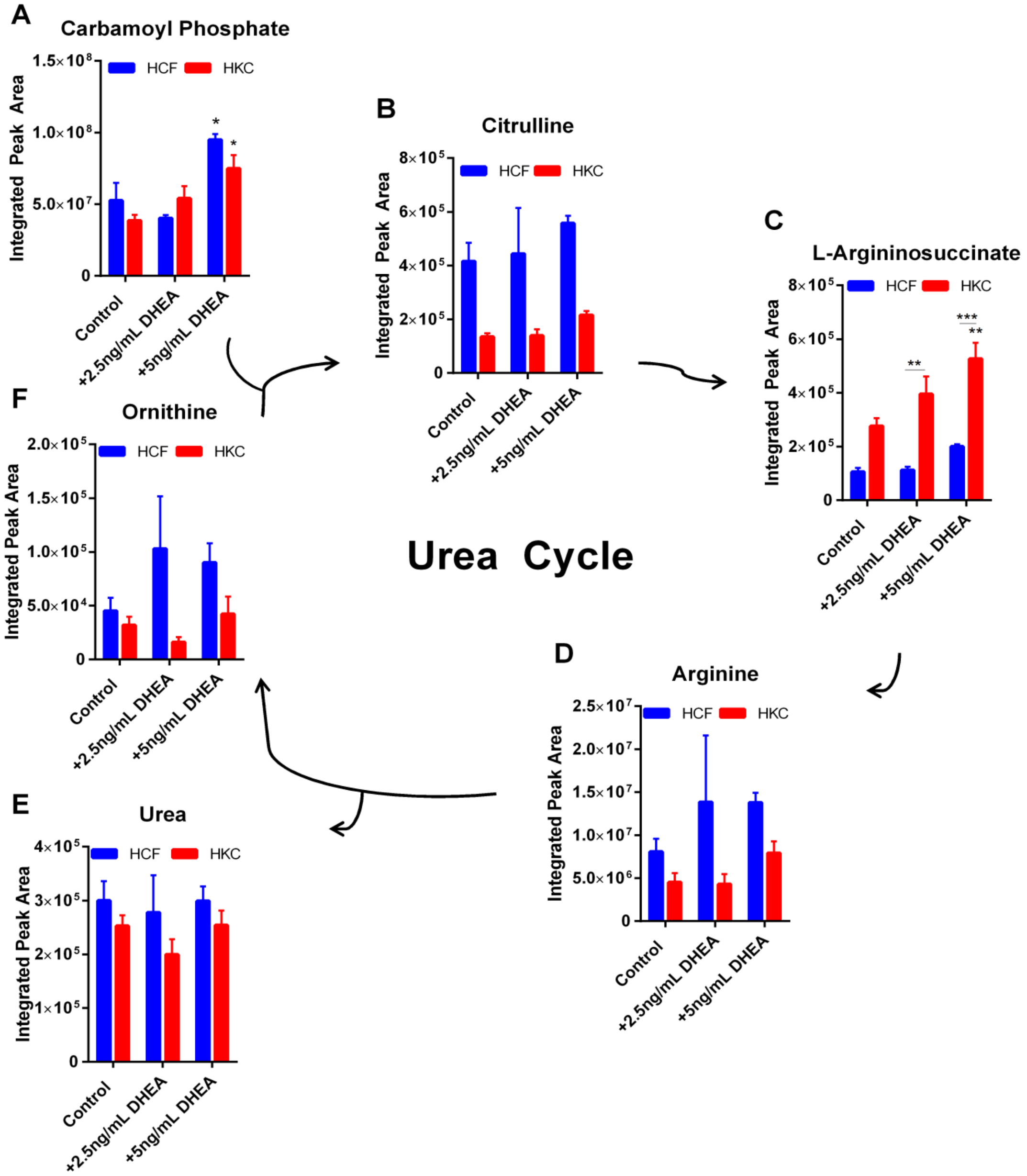

Supplemental Figure 2. Increased urea cycle flux with DHEA treatment in both HCFs and HKCs. The urea cycle is a major metabolic regulator of arginine metabolism, which serves as a precursor to proline, the core amino acid that makes up collagen monomers. HKCs showed a substantial reduction in arginine levels untreated with a significant increase in L-argininosuccinate with DHEA treatment (2.5ng/mL and 5ng/mL) in HKCs, but not HCFs. n=3, error bars represent standard error of the mean. Statistical significance was determined by ANOVA. *p≤0.05, **p≤0.01, ***p≤0.001.

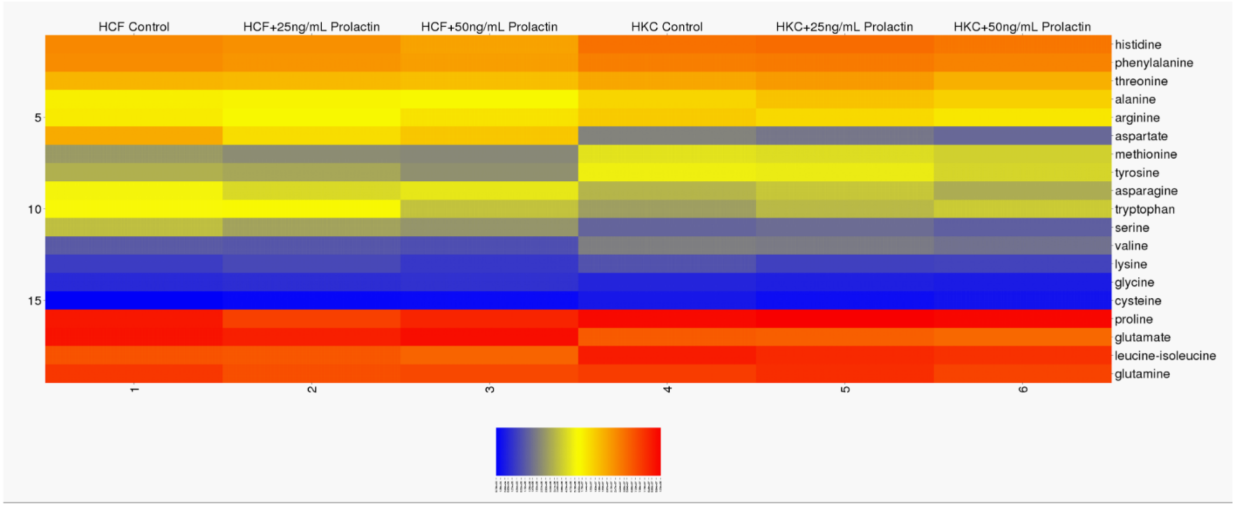

Supplemental Figure 3. Heat map of amino acid flux with increasing concentration of prolactin (25ng/mL and 50ng/mL) in HCFs and HKCs. Compared to the effects of DHEA and 17β-estradiol on amino acid flux, prolactin did not significantly modulate protein degradation or assembly.

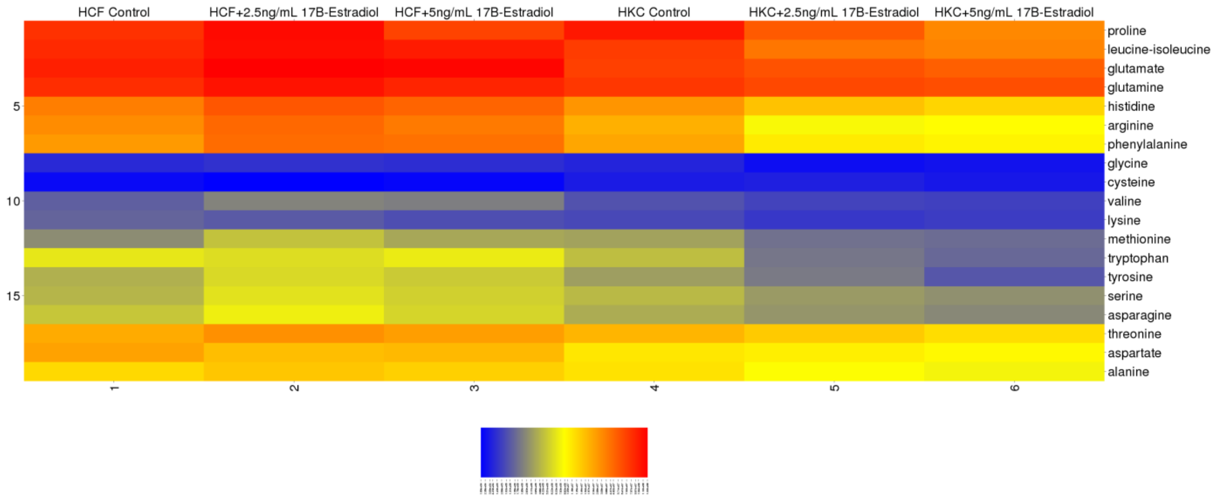

Supplemental Figure 4. Heat map of free amino acid flux in HCFs and HKCs with increasing concentrations of 17β-estradiol (2.5ng/mL and 5ng/mL). The blue color indicates metabolites at low concentrations with a transition to yellow and orange representing higher concentrations.

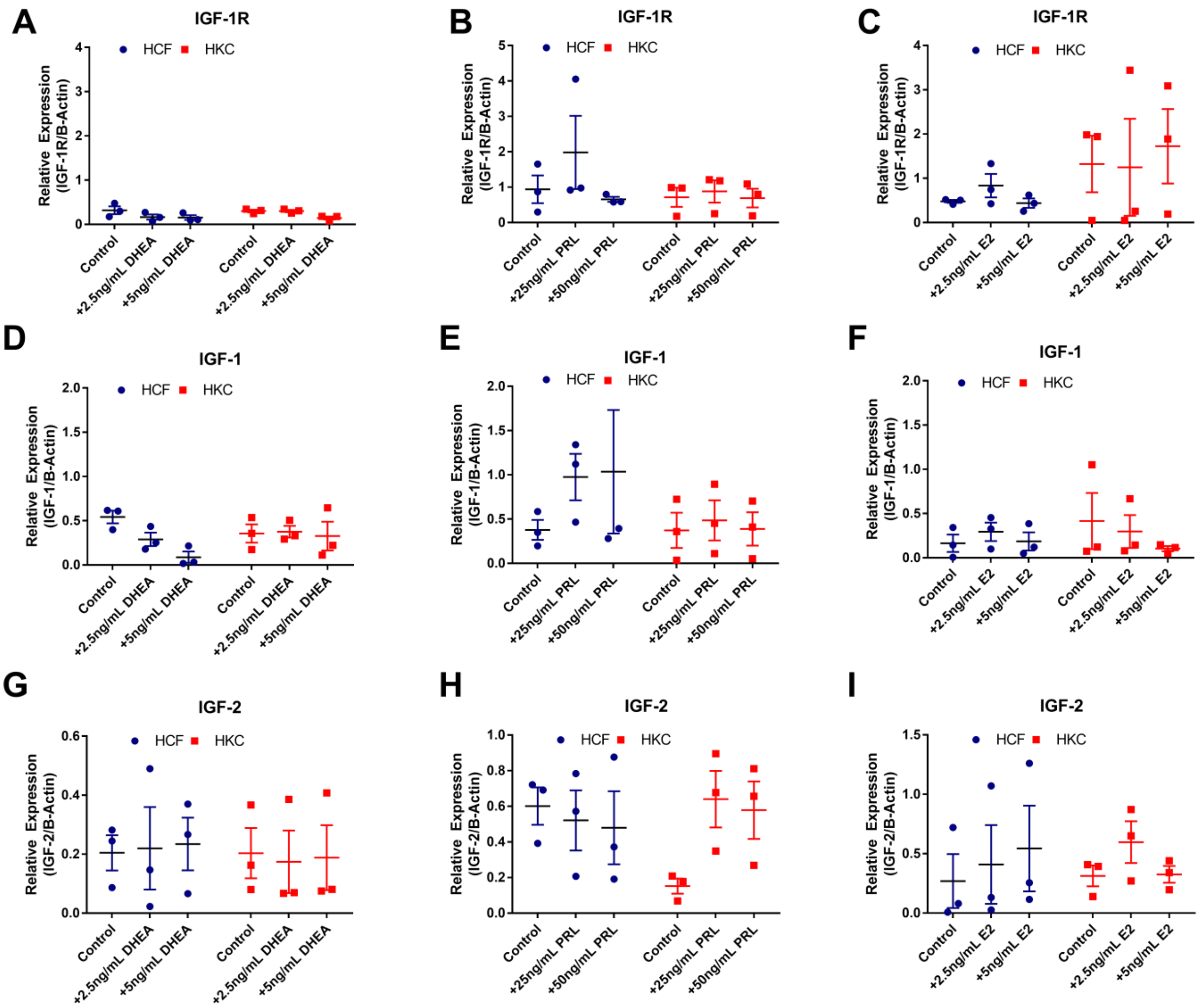

Supplemental Figure 5. Relative protein expression of (A-C) IGF-1R, (D-F) IGF-1, and (G-I) IGF-2 in HCFs and HKCs.
